# The impact of phrasing on advice-taking under gain and loss frames in a reinforcement learning paradigm

**DOI:** 10.3389/fpsyg.2025.1693546

**Published:** 2025-12-17

**Authors:** Xuanhan Chang

**Affiliations:** 1 King’s College London, Institute of Psychiatry, Psychology and Neuroscience, London, United Kingdom; 2 School of Psychology, South China Normal University, Guangzhou, China

**Keywords:** advice taking, social learning, reinforcement learning, computational modeling, framing effect

## Abstract

**Introduction:**

Grounded in Behrens et al.’s (2008) advice-taking paradigm, this study investigates how advice phrasing (positive vs. negative) and task framing (gain vs. loss) influence the extent to which individuals integrate advice during decision-making. Rather than focusing on isolated choice outcomes, we examined the cognitive processes underlying advice use through a reinforcement learning (RL) framework.

**Methods:**

Across two experiments (*N* = 38 and *N* = 74), participants completed probabilistic decision-making tasks while receiving trial-by-trial advice. Computational modeling was used to estimate the latent advice reference weight (*ω*), reflecting reliance on advice throughout the learning process, as well as the advice-specific learning rate (*α*_*a*_). Behavioral measures of advice-taking (advice–choice consistency) were analyzed alongside modeling-derived parameters.

**Results:**

Both behavioral indices and parameter estimates showed that participants relied more on positively phrased advice than negatively phrased advice. Moreover, advice phrasing interacted with task framing: positively phrased advice exerted a stronger influence under the gain frame, whereas negatively phrased advice was more influential under the loss frame. This interaction was robustly captured by the modeled advice-weight parameter (ω), although not consistently evident in behavioral choice patterns. Modeling results further showed that the advice-specific learning rate (*α*_*a*_) was significantly higher for positively phrased advice, suggesting greater updating from such information.

**Discussion:**

These findings provide a mechanistic understanding of how social (advice phrasing) and contextual (task framing) features jointly shape advice integration and inform more effective communication strategies in decision-making contexts.

## Introduction

In everyday life, people often seek advice from others when making decisions—whether it involves financial investments, medical treatments, or daily choices. Integrating such advice can enhance decision quality by combining personal knowledge with external expertise (Minson et al., 2011; Gino and Schweitzer, 2008). The field of advice-taking has identified several key factors influencing whether and how people follow advice: the characteristics of the advisor (Gino et al., 2009), the traits of the advisee (Gino et al., 2012), the content and phrasing of the advice (Piray et al., 2021), and the context of the decision-making task (Ache et al., 2020).

Among these, two features have received increasing attention: advice phrasing and task framing. “Advice phrasing” refers to how the recommendation is worded—for example, in a positive (appetitive) form (“Choose the green option; you will receive a reward”) versus a negative (aversive) form (“Choose the blue option; you will incur a loss”). Prior work suggests that positively phrased advice tends to be perceived as more persuasive and trustworthy than negatively phrased advice (Piray et al., 2021). Neuroimaging evidence further indicates that negatively framed advice may be discounted at the neural level, with individuals placing more weight on their own experiences when processing aversive advice (Piray et al., 2021). Task framing, on the other hand, refers to how the decision outcomes are structured—often in terms of gains versus losses.

Previous studies have indicated that the task frame can shift individuals’ preferences: individuals tend to exhibit risk aversion in a gain context but become more risk-seeking in a loss context (Kahneman and Tversky, 1979). These two features—advice phrasing and task framing—have typically been studied in isolation, even though, in real-world scenarios, advice is often embedded within a broader motivational context or “frame.” Understanding how phrasing and framing interact is therefore essential for explaining advice-taking in more ecologically valid settings. Indeed, decision framing may modulate the cognitive and motivational processing of advice. Research on regulatory focus suggests that gain-framed contexts evoke a promotion focus (an approach-oriented motivation), whereas loss-framed contexts evoke a prevention focus (an avoidance-oriented motivation) (Lee and Aaker, 2004; Motyka et al., 2014).

People tend to be more persuaded by messages that align with their current motivational focus: positively phrased messages are more effective under a promotion focus, whereas negatively phrased messages are more effective under a prevention focus (Bosone et al., 2015). In other words, the impact of advice phrasing might be amplified when it matches the decision’s frame. This perspective leads us to expect that positive phrasing could be especially persuasive in gain contexts, whereas negative phrasing might be more influential in loss contexts.

Prior studies of advice-taking have predominantly relied on binary outcome measures—such as whether participants follow advice on each trial—to assess advice-taking (De Hooge et al., 2014; Du et al., 2022). While such measures capture behavioral alignment, they fail to reveal the internal cognitive processes by which advice is evaluated and integrated, particularly in uncertain environments. To address this, we adopt a computational modeling approach to uncover the latent mechanisms of advice use.

Recent research suggests that advice-taking involves the integration of both personal and social sources of information; these are two distinct but interacting learning processes: (1) self-learning, where individuals update beliefs about the environment based on trial-by-trial outcomes, and (2) advice learning, where they update beliefs about the advisor’s reliability (Piray et al., 2021). Final decisions reflect the subjective weight assigned to each source of information (Soll and Mannes, 2011; Bednarik and Schultze, 2015). Reinforcement learning (RL) models are well suited to capturing both processes, as they formalize how individuals update value representations over time in response to feedback (Piray et al., 2021).

Behrens et al. (2008) and Piray et al. (2021) employed an advice-taking paradigm in which participants made repeated choices between two options (e.g., cards) on each trial while receiving advice about which option was likely to yield a reward. The reward probabilities associated with each option changed unpredictably over time, requiring participants to learn the currently optimal choice through trial-by-trial feedback. This design allowed researchers to examine how individuals integrate social (advice) and experiential (feedback) information under conditions of uncertainty. While the present study adopts a similar probabilistic learning task structure, its primary focus diverges from that of Piray et al. (2021).

Specifically, Piray aimed to investigate how individuals dynamically learn and track the reliability of an advisor whose accuracy fluctuates over time. In contrast, our study centers on how advice phrasing interacts with decision framing (gain vs. loss) to influence the reference weight assigned to advice. (1) We implemented two task frames—a gain frame, in which correct choices yield a reward, while incorrect choices result in no loss, and a loss frame, in which incorrect choices result in a loss, while correct choices yield no reward. Within each frame, we embedded two types of advice phrasing (see Methods), allowing us to systematically examine the interaction between phrasing and motivational context. This design extends Piray et al. (2021), whose paradigm focused on advice phrasing but did not manipulate task framing, thus limiting the ability to investigate how these two factors jointly shape advice-taking. (2) We held adviser reliability constant across trials in order to isolate the effects of advice phrasing and decision framing from the influence of dynamic advisor learning.

Building on Ez-zizi et al. (2023), we further distinguish between two types of uncertainty that influence learning dynamics: expected uncertainty (i.e., the uncertainty inherent in the reward probability itself, such as stochastic feedback even in a stable environment) and unexpected uncertainty (i.e., volatility, where the environment changes unexpectedly, requiring belief updating). Our task structure embeds both forms of uncertainty: even when the reward probability is stable within a block (expected uncertainty), participants must also monitor for unsignaled changes across blocks (unexpected uncertainty). This dual-uncertainty structure places significant demands on learners and is well suited for testing how people integrate advice during ongoing learning.

Although previous work has shown that people adapt their learning rate to environmental volatility (Behrens et al., 2007) and that this can be modeled using Bayesian delta-rule RL models (Nassar et al., 2010), recent findings suggest that simpler models may be more effective under such conditions. Ez-zizi et al. (2023) found that in these environments, simple RL models outperformed Bayesian RL models, suggesting that humans may adopt heuristically efficient learning strategies when tracking hidden states.

Following this line of research, we employed a simple delta-rule RL model to capture participants’ trial-by-trial updates of advice and self-related value estimates. To estimate individual-level parameters robustly, we used hierarchical Bayesian modeling, which pools information across participants to improve estimation stability and generalizability (Zhang et al., 2020). Here, we use “Bayesian” to denote hierarchical estimation of model parameters rather than treating Q-values as probabilistic variables, as in fully Bayesian RL frameworks (e.g., Ez-zizi et al., 2015). Our approach aligns more closely with previous cognitive modeling studies using Bayesian inference to recover individual-level parameters in psychological tasks (Zhang et al., 2020). It is particularly well-suited to our aim: estimating a latent advice weight that captures how strongly participants integrate advice across different phrasing and framing manipulations.

This study has two primary aims. First, at the behavioral level, we investigate how advice phrasing and task framing interact to shape advice-taking behavior. Second, we use a computational modeling approach to quantify the reference weight (𝜔) participants assign to advice. We hypothesize that at both the behavioral and computational levels: (1) positively framed advice will be more persuasive than negatively framed advice; (2) the effectiveness of advice phrasing will depend on task framing, with positive phrasing being more persuasive in gain contexts and negative phrasing being more persuasive in loss contexts.

## Experiment 1: impact of phrase on advice taking

### Materials and methods

#### Participants

Using G * power 3.1 (Faul et al., 2009), we conducted *a priori* power analyses to determine the required sample size for experiment 1. We based our calculation on a within-subject design. Given a medium-sized effect (*f* = 0.25) and *α* = 0.05, with power (1−β) = 0.80, we required at least 34 participants. We recruited participants by posting information online within South China Normal University. The participants were required to have normal or corrected-to-normal vision. A total of 38 eligible participants were recruited, including 24 women and 14 men. All were university students aged 18–25, and none had previously participated in such experiments. Ethical approval for this study was obtained from the Human Research Ethics Committee for Non-Clinical Faculties, School of Psychology, South China Normal University. Written informed consent was obtained from all participants.

#### Design

Experiment 1 employed a within-subject design with a single factor: advice phrasing (positive vs. negative).

#### Procedure

We used MATLAB R2019a to program the behavioral experiments. Referring to the advice-taking paradigm used in the study by Behrens et al. (2008), participants acting as players need to evaluate the probability of winning for the two cards (green and blue) and then make a choice between them. Before choosing a card, an adviser assigned by the program will provide advice. The advice is presented in a sentence. Positively expressed advice focuses on positive outcomes, while negatively expressed advice focuses on negative outcomes.

For example, to provide the same advice of choosing the green card and avoiding the blue card, a positively expressed advice would be “Choose the green card; you will receive a reward,” while a negatively expressed advice would be “Choose the blue card; you will incur a loss” (detailed information about the advice assignment can be found in the Supplementary material). Each time a correct choice is made, the participant will receive a reward. If an incorrect choice is made, the participant will incur a loss. The basic participant fee is 10 yuan, with an additional 5 yuan determined by the amount of money accumulated (or remaining) after the experiment.

**Figure 1:**
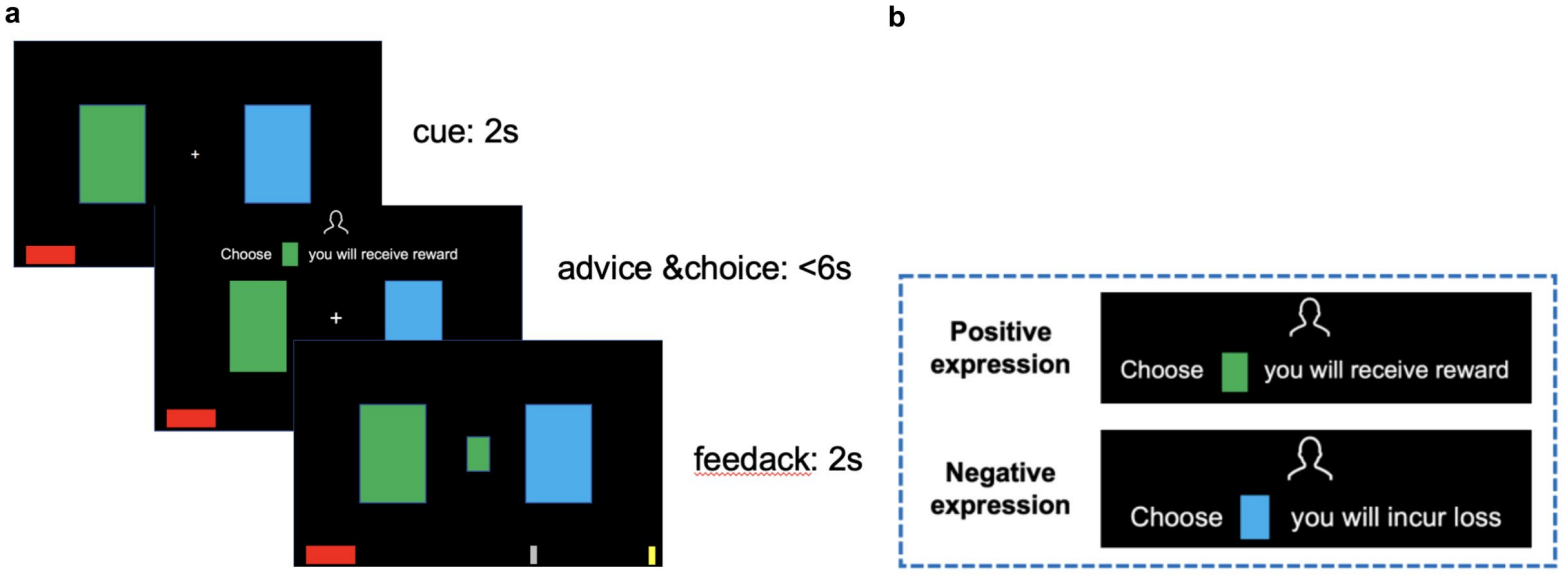
Task procedure and examples of advice phrasing used in Exp 1. **(a)** Timeline of the experimental task. On every trial, participants are presented with a cue and then receive written advice from the adviser. After choosing (button press) between two decision options, the correct choice (feedback) is revealed, and the accumulated money (red bar at the bottom) increases with each correct response or decreases with each incorrect response. **(b)** Advice examples in Exp 1. Advice in positive expression focuses on positive results, such as “Choose a card, you will receive a reward,” and advice in negative expression focuses on negative results, such as “Choose a card, you will incur loss.”

Before the experiment begins, instructions (see Supplementary material) are presented to inform participants about the upcoming task, emphasizing that correct choices will be rewarded and incorrect choices will incur a loss. Participants will be told that the adviser in the experiment possesses more information and will provide advice; their advice may sometimes be incorrect, but their overall accuracy is greater than 50% (similar to real investment consulting scenarios, where consultants have a higher accuracy rate due to their expertise). A red bar at the bottom of the screen indicated the participant’s accumulated performance. Each time a correct choice is made, the bar will lengthen; conversely, it will shorten with each incorrect choice. Two vertical silver markers along the bar served as progress thresholds (not a timeline). When the red bar extended beyond the right silver marker, it indicated that the participant had made a sufficient number of correct choices to reach a threshold, and their bonus payment increased by 3 yuan; when it shortened to the silver marker on the left, the participant’s bonus payment would decrease by 2 yuan. When the red bar reaches the far-right end (or shortens to disappear), the payment will increase by 5 yuan (or decrease by 5 yuan). The practice experiment includes 10 trials, followed by the formal experiment. The experimental task is shown in Figure 1. A fixation point (+) will appear in the center of the screen, with green and blue cards on either side. After 2 s, the advice was presented for 6 s, during which participants should make their choice. After making a choice, they will receive feedback for 2 s.

**Figure 2:**
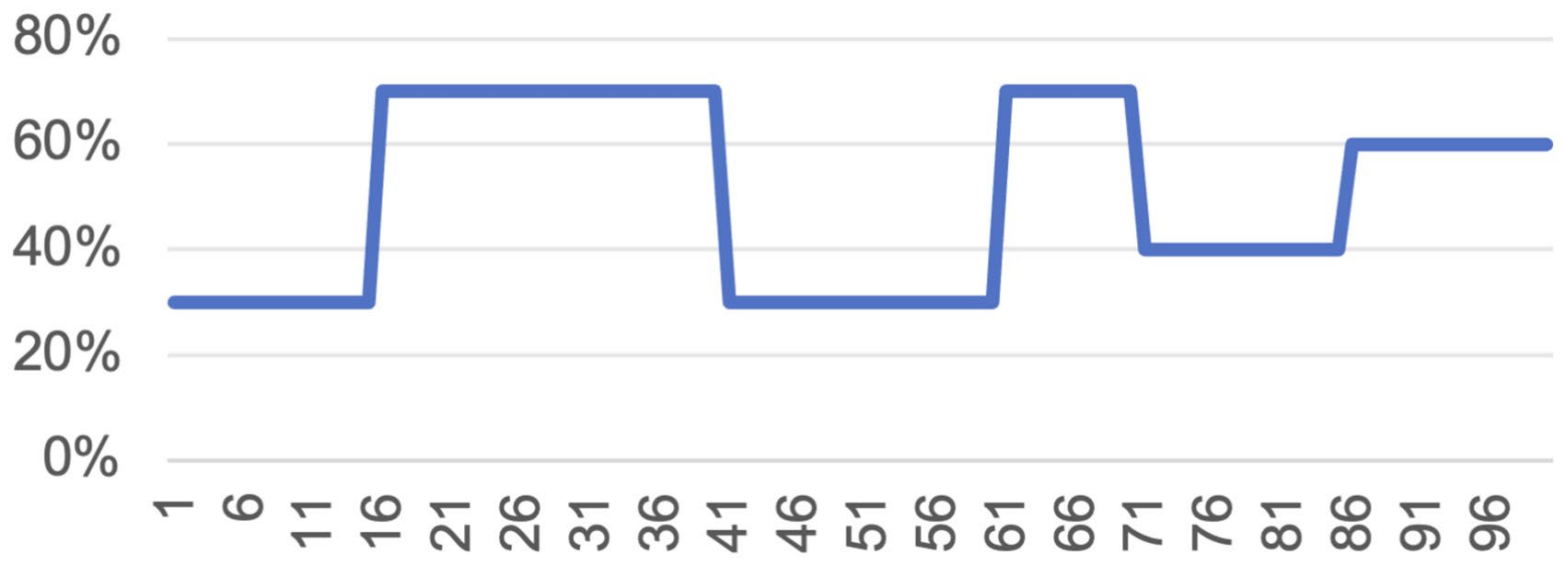
Probability of the green card being correct.

The formal experiment consists of 100 trials. The “winning” probability of the green card (see Figure 2) was determined by the program and alternated across blocks (e.g., 70 to 30%) to induce environmental volatility and encourage belief updating, consistent with prior paradigms (Behrens et al., 2008; Piray et al., 2021). The accuracy of the adviser’s suggestions was held constant at 60% across trials. This level was chosen based on pilot testing: higher accuracies (e.g., 70%) led participants to rely almost exclusively on the advice, whereas lower rates discouraged advice use entirely. A 60% accuracy helped balance advice-taking with self-learning. Importantly, this fixed accuracy allowed us to isolate the effects of advice phrasing and task framing, without confounding influences from dynamic trust learning. This design choice was further intended to approximate real-world situations in which the external environment may shift over time, but the perceived reliability of advisers (e.g., doctors, consultants) remains relatively stable. We emphasize that this analogy is conceptual rather than empirical—we do not claim a specific accuracy level for real-life consultants.

#### Computational modeling

In this advice-taking task, participants need to simultaneously consider others’ advice and information based on their own experience, both of which are independently set before the experiment. Therefore, to make optimal decisions, participants need to learn from each feedback: (1) the probability of the advice being correct (advice-learning); (2) the probability of the card “winning” (self-learning); (3) combining these two probabilities to make a choice. To simulate the three processing steps of the participants, the basic idea of this study’s modeling is to use two reinforcement learning models to simulate the first two learning processes, then combine them using the advice reference weight.

Reinforcement Learning (RL) (Sutton and Barto, 2018) models are widely used in feedback-based probabilistic learning tasks to uncover the underlying decision variables in learning and decision-making tasks (Zhang et al., 2020). In these learning tasks, participants’ choices are influenced by environmental feedback, which in turn allows them to update the values of previously chosen options. Classical RL models typically assume that learners only update the value of the currently chosen option. However, some researchers have proposed an alternative RL model, known as the Fictitious Reinforcement Learning model (Hampton et al., 2008; Gläscher et al., 2009). This model assumes that participants simultaneously update the values of both the chosen and unchosen options. This model has been shown to fit better than the basic RL model in various learning tasks (Hampton et al., 2008; Hill et al., 2017). Hereafter, this model will be referred to as Fict+RL. Additionally, some researchers have proposed that participants exhibit asymmetric learning rates during the learning process, meaning that learning rates differ when the prediction error is positive versus negative (Frank et al., 2007; Gershman and Niv, 2015; Lefebvre et al., 2017). Hereafter, this model will be referred to as RL±.

This study uses eight different models (see Table 1). These models can be divided into four categories: basic RL models, Fict+RL models, RL ± models, and Full models. The basic RL model, namely the ChoiceRL_AdviceRL model, uses two simple RL models to separately simulate the participant’s self-learning and advice-learning. The cognitive models (Equations 1, 2) are as follows:


$$V{\left(i\right)}_{\left(t+1\right)}^{c}=V{\left(i\right)}_{t}^{c}+\alpha^{c}\cdot (R_{t}^{c}-V{\left(i\right)}_{t}^{c})$$ (1)

$$V{\left(i\right)}_{\left(t+1\right)}^{a}=V{\left(i\right)}_{t}^{a}+\alpha^{a}\cdot (R_{t}^{a}-V{\left(i\right)}_{t}^{a})$$ (2)

**Table 1:** Model comparison of experiment 1.

Categories	Model	Name	LOOIC
Basic RL	M1	ChoiceRL_AdviceRL	5334.1
Fict+RL	M2a	ChoiceFict_AdviceRL	5230.7
	M2b	ChoiceRL_AdviceFict	5230.1
	M2c	ChoiceFict_AdviceFict	5106.4
RL±	M3a	ChoiceRL±_AdviceRL	5052.0
	M3b	ChoiceRL_AdviceRL±	5314.9
	M3c	ChoiceRL±_AdviceRL±	5290.6
Full	M4	ChoiceFictRL±_AdviceFictRL	4836.8

Here, $V{\left(i\right)}_{t}^{c}$ represents the expected value for card i (green or blue) in trial t based on self-learning. $V{\left(i\right)}_{t}^{a}$ represents the expected value for advice content i (follow or unfollow) in trial t based on advice-learning. The expected value in trial t + 1 ( $V{\left(i\right)}_{t+1}$ ) will be updated based on the difference between the reward ( $R_{t}$ ) and the previous expectation ( $V{\left(i\right)}_{t}$ ). This difference is called the prediction error. The impact of the prediction error on forming a new expectation is determined by the learning rate ( 0<α<1 ). The learning rate is one of the most important free parameters in RL models; a higher value indicates that new information has a greater impact on future behavior compared to previous information.

For self-learning, the reward ( $R_{t}^{c}$ ) was coded as 1 when the participant’s choice was correct, and −1 when it was incorrect. The initial expected values for both cards ( $V{\left(i\right)}_{1}^{c}$ ) were set to 0 at the start of the task. For advice-learning, the reward ( $R_{t}^{a}$ ) was coded as 1 when the advice was correct, and −1 when it was incorrect. The initial expected values for advice content ( $V{\left(i\right)}_{1}^{a}$ ) were set to 0 at the start of the task.

Choice probability $P_{t}$ is determined by the following softmax function. Functions (Equations 3–5) are choice models:


$$P{\left(C=G\right)}_{t}^{c}=\frac{1}{1+e^{-\tau^{c}*(V{\left(G\right)}_{t}^{c}-V{\left(B\right)}_{t}^{c})}}$$ (3)

$$P{\left(C=F\right)}_{t}^{a}=\frac{1}{1+e^{-\tau^{a}*(V{\left(F\right)}_{t}^{a}-V{\left(U\right)}_{t}^{a})}}$$ (4)

where $P{\left(C=G\right)}_{t}^{c}$ is the probability of choosing a green card based on self-learning, and $P{\left(C=F\right)}_{t}^{a}$ represents the probability of choosing the option recommended by the adviser, based on advice-learning. $V{\left(G\right)}_{t}^{c}$ and $V{\left(B\right)}_{t}^{c}$ represent the expected values for green card and blue card, respectively, while $V{\left(F\right)}_{t}^{a}$ and $V{\left(U\right)}_{t}^{a}$ represent the expected value for choosing the option recommended by the adviser, respectively. The inverse temperature $\tau^{c}$ and $\tau^{a}\;$ determines the sensitivity of the choice probabilities to the difference between the expectation values. These two probabilities are then combined to determine the final choice probability:


$$P{\left(C=G\right)}_{t}=\beta \cdot \omega \cdot P{\left(C=F\right)}_{t}^{a}+(1-\omega )\cdot P{\left(C=G\right)}_{t}^{c}$$ (5)

where $P{\left(C=G\right)}_{t}$ is the final probability of choosing a green card. ω is the advice reference weight, while ( 1−ω) is the tendency to make a choice solely based on self-experience. β equals 1 when the advice is to choose a green card; otherwise, β equals −1.

For the remaining three types of models, their cognitive models are detailed in the Supplementary material: Construction of models; their choice models are the same as M1.

We used rstan2.34 (Stan Development Team, 2024) for model construction and data fitting. The models are fitted using the Monte Carlo Markov Chain (MCMC) sampling method to obtain posterior parameter estimates. Each model includes four Monte Carlo Markov chains, with each chain iterating 4,000 times. The first half of the iterations is used for warming up and is not included in the final analysis. All Bayesian models passed model diagnostics, with the parameter convergence index (Gelman-Rubin convergence index) being less than 1.01.

To estimate individual-level parameters, we employed hierarchical Bayesian modeling implemented in RStan. The learning rate ( α ), softmax temperature (*τ*), and advice weight (*ω*) were modeled as individual-level parameters drawn from group-level distributions. Specifically, we used weakly informative priors:

Group-level means:

$$\mu_{\alpha }\sim \text{Uniform}(0,1)$$

$$\mu_{\tau }\sim \text{Uniform}(0,3)$$

$$\mu_{\omega }\sim \text{Uniform}(0,1)$$

Group-level standard deviations:

$$\sigma_{\alpha }\sim \text{halfCauchy}(0,1)$$

$$\sigma_{\tau }\sim \text{halfCauchy}(0,3)$$

$$\sigma_{\omega }\sim \text{halfCauchy}(0,1)$$

Individual-level parameters:

$$\alpha_{i}\sim \text{Normal}{\left(\mu_{\alpha },\sigma_{\alpha }\right)}_{\Gamma (0,1)}$$

$$\tau_{i}\sim \text{Normal}{\left(\mu_{\tau },\sigma_{\tau }\right)}_{\Gamma (0,3)}$$

$$\omega_{i}\sim \text{Normal}{\left(\mu_{\omega },\sigma_{\omega }\right)}_{\Gamma (0,1)}$$

These priors were chosen to reflect plausible parameter ranges while allowing sufficient flexibility during estimation. All prior settings were consistent across models and experiments.

#### Statistical analysis

For the behavioral data, a repeated-measures ANOVA was conducted to investigate whether there is a difference in task accuracy and advice-choice consistency rate between positive and negative phrasing. For the model-based data, we compared the advice reference weight (ω) and advice learning rate (α) across advice phrasing conditions using paired-sample *t*-tests, as each parameter was estimated per condition per participant.

### Results

#### Behavioral results

As a control analysis, we examined whether participants exhibited a color preference in their choices. Across all trials and participants (including Exp 1 and Exp 2), the green card was chosen on 48.47% of trials, suggesting no systematic bias due to color associations.

*Accuracy*: The results showed that there was no significant difference between the two phrasing conditions (*F*(1,37) = 1.53, *p* = 0.224, η^2^ = 0.040, 95% CI [0.00, 0.17]). Participants’ accuracy was similar for positively phrased advice (M = 0.57, 95% CI [0.55, 0.59]) and negatively phrased advice (M = 0.54, 95% CI [0.52, 0.57]), indicating that the phrase of the advice had no effect on participants’ task performance.

**Figure 3:**
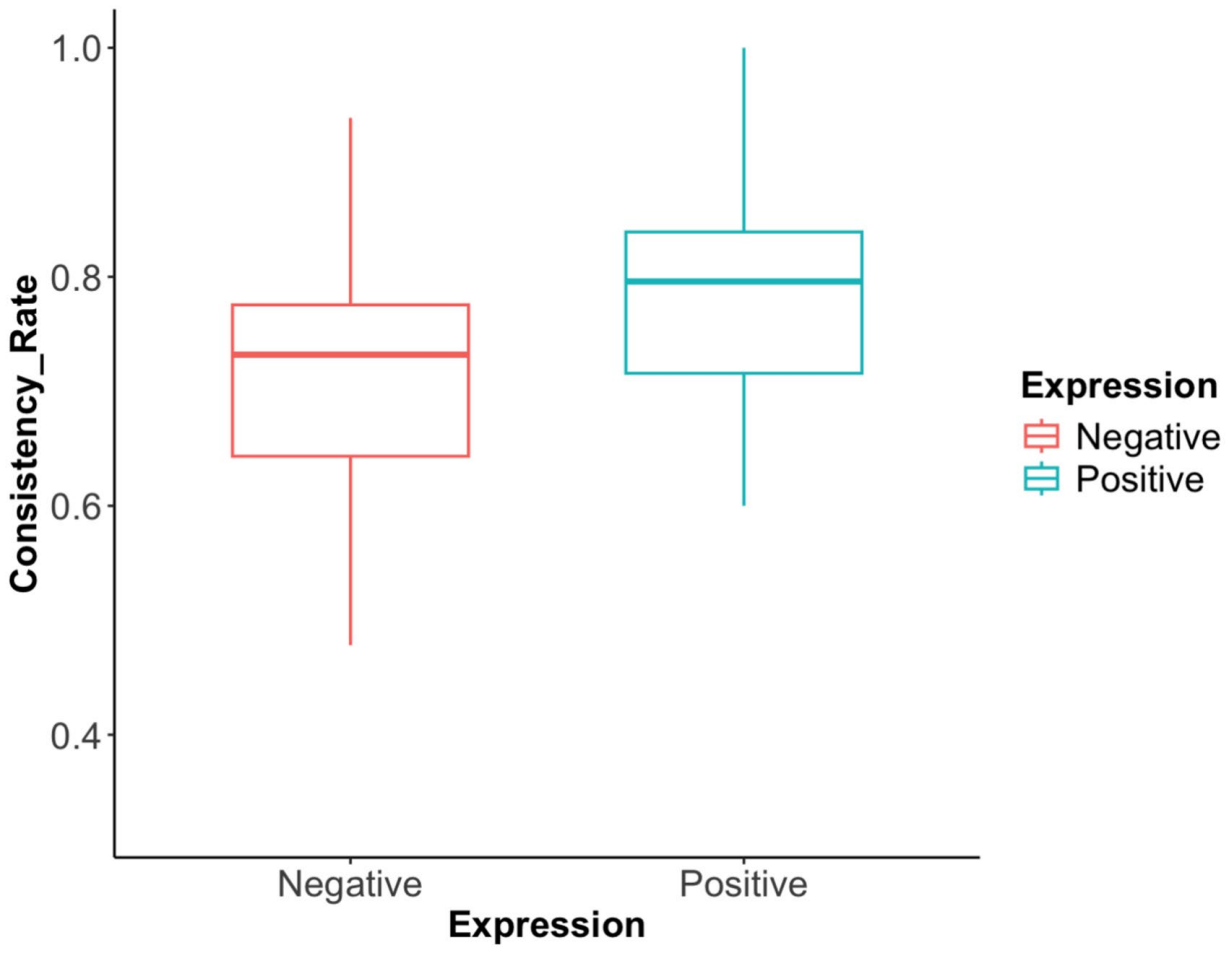
Advice-choice consistency rate in Exp 1. The advice-choice consistency rates under positive expression and negative expression were depicted. The consistency rate for positively expressed advice is significantly higher than that for negatively expressed advice.

*Advice-choice consistency rate*: The results (see Figure 3) showed that the mean advice-choice consistency rate was significantly higher for positively phrased advice (M = 0.78, 95% CI [0.74, 0.81]) than for negatively phrased advice (M = 0.70, 95% CI [0.65, 0.75]). This difference was statistically significant (F(1,37) = 8.16, *p* = 0.007, η^2^ = 0.181, 95% CI [0.02, 0.42]), indicating that advice phrasing had a substantial effect on the degree to which participants’ choices aligned with the advice.

#### Modeling results

*Model comparison*: We used the R package loo (Vehtari et al., 2017) to calculate the model comparison metric: Leave-One-Out Information Criterion (LOOIC). This metric reflects the model’s ability to explain the current data and predict unknown data. The smaller the LOOIC value, the better the model’s performance. The results (see Table 1) showed that among the Fict+RL models, the ChoiceFict_AdviceFict model had the smallest LOOIC value. Among the RL ± models, the ChoiceRL±_AdviceRL model had the smallest LOOIC value. Consequently, we considered adding an eighth model, the ChoiceFictRL±_AdviceFictRL model, and calculated its LOOIC value. We found that it had the smallest LOOIC value among all models, indicating that this is the optimal model.

The model names are based on the types of RL models used to simulate two learning processes (self-learning and advice-learning).

**Figure 4:**
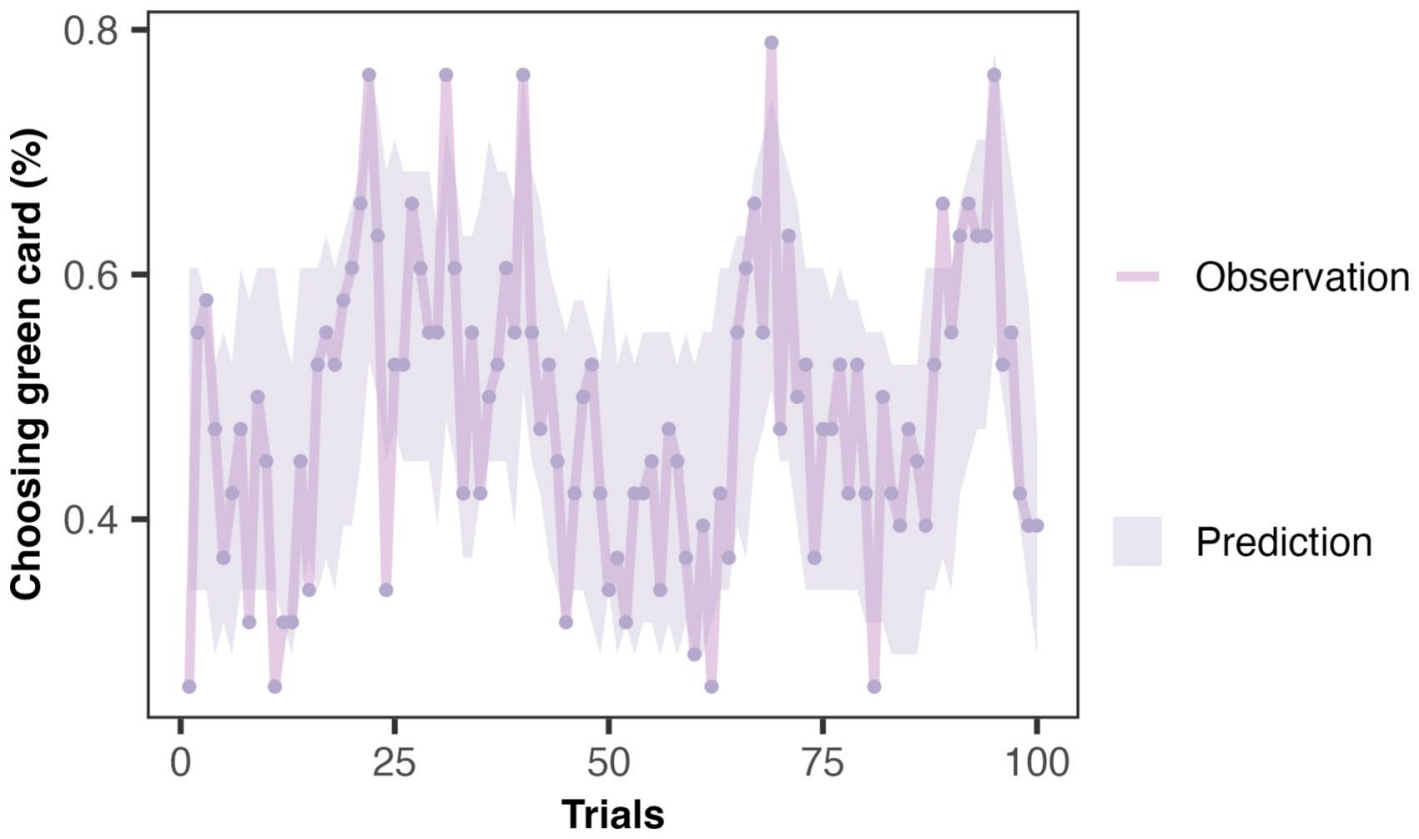
Posterior predictive check of winning model in Exp 1. The dots in the figure represent the actual behavioral data for each trial (the average probability that subjects chose the green card), while the shaded area represents the simulated data generated by the winning model (with a 95% credible interval). The high overlap between the two indicates that M4 can capture the subjects’ behavioral characteristics.

*Model validation*: Even though the model comparison results indicate that M4 is the optimal model, it remains unclear whether M4 can capture the characteristics of the real data. Therefore, we used a trial-by-trial Posterior Predictive Check (PPC) to validate the predictive effectiveness of M4 on behavioral data. This method is the most widely used model validation method (Gelman et al., 2013; Zhang et al., 2020). PPC uses the posterior distribution of parameters obtained from parameter estimation to generate new predicted datasets and compares whether these predicted data can adequately explain the observed data. As shown in Figure 4, the shaded area represents the credible interval (95% credible interval) of the predicted data distribution. The high overlap between the predicted and actual data indicates that M4 can accurately capture participants’ behavioral characteristics.

*Parameter analysis*: We used the posterior mean of the winning model’s parameters to conduct further data analysis. The mean is used instead of the mode because, in Markov Chain Monte Carlo (MCMC), especially in Hamiltonian Monte Carlo (HMC) as implemented in Stan, the mean is much more stable than the mode and can serve as a point estimate for the entire posterior distribution (Gelman et al., 2013; Zhang and Gläscher, 2020). This study primarily focuses on the advice reference weight ω and the learning rate $\alpha^{a}$ in advice-learning.

**Figure 5:**
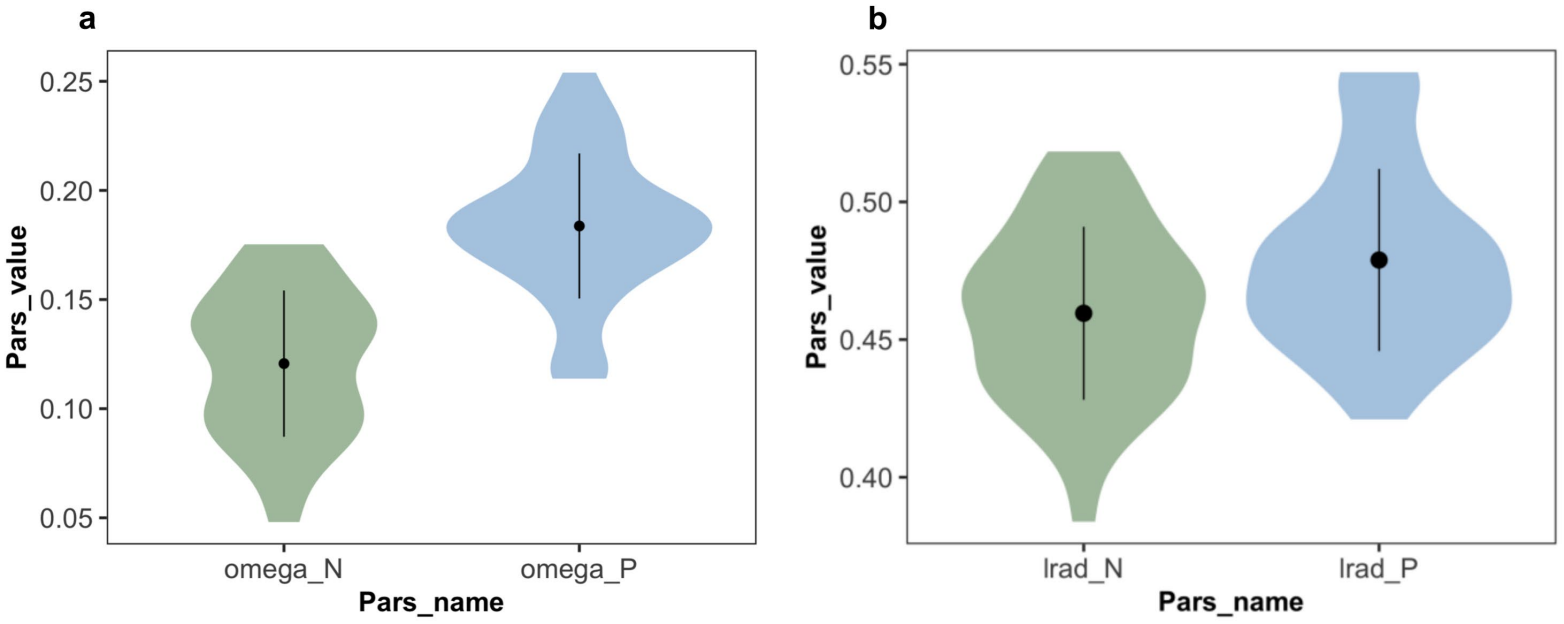
Estimated parameter values in Exp 1. **(a)** Weight parameters from the winning model, in which omega_P and omega_N represent the weight ω that subjects employed advice on trials associated with positive and negative expression. Participants relied more on the positively expressed advice than on the negatively expressed advice. **(b)** Learning rate parameters from the winning model, in which lrad_P and lrad_N represent the learning rate $\;\alpha^{a}$ of advice being correct associated with positive and negative expressions. The learning rate of positive expression is relatively higher than that of negative expression.

The statistical analysis results (see Figure 5a) showed that the advice reference weight under negative expression (M = 0.12, 95% CI [0.11, 0.13]) was significantly lower than that under positive expression (M = 0.18, 95% CI [0.17, 0.20]) (t (37) = −7.94, *p* < 0.001, dz. = 1.288, 95% CI [0.84, 1.73]). The t-test results for advice-taken learning rate $\alpha^{a}$ (see Figure 5b) showed that the learning rate under positive expression (M = 0.46, 95% CI [0.45, 0.47]) was significantly higher than under negative expression (M = 0.48, 95% CI [0.47, 0.49]) (t (37) = 2.61, *p* = 0.010, dz. = 0.424, 95% CI [0.08, 0.77]). This indicates that the advice phrase has a significant impact on both the advice reference weight and the learning rate in advice-learning.

## Experiment 2: impact of advice phrasing and task frame interaction on advice taking

### Methods

#### Participants

Using G*Power 3.1 (Faul et al., 2009), we also conducted an *a priori* power analysis for Experiment 2, which used a mixed design with one within-subject factor (advice phrasing: positive vs. negative) and one between-subject factor (framing: gain vs. loss). Assuming a medium effect size (*f* = 0.25), *α* = 0.05, and power (1–β) = 0.80, the analysis indicated that a minimum of 34 participants (17 per group) would be required to detect interaction effects. Our final sample size (*n* = 39; 19 in the gain frame, 20 in the loss frame) met this requirement. We recruited participants by posting information online within South China Normal University. The participants were required to have normal or corrected-to-normal vision. A total of 40 eligible participants were recruited, with one participant excluded due to an accuracy rate below 40%. The remaining 39 participants met the criteria, including 26 females and 13 males, all university students aged 18–27, none of whom had previously participated in similar experiments.

#### Design

We use a 2 (advice phrasing: positive, negative) × 2 (task frame: gain, loss) mixed-design experiment. The task frame was a between-subjects variable, and the advice phrasing was a within-subjects variable.

#### Procedure

**Figure 6:**
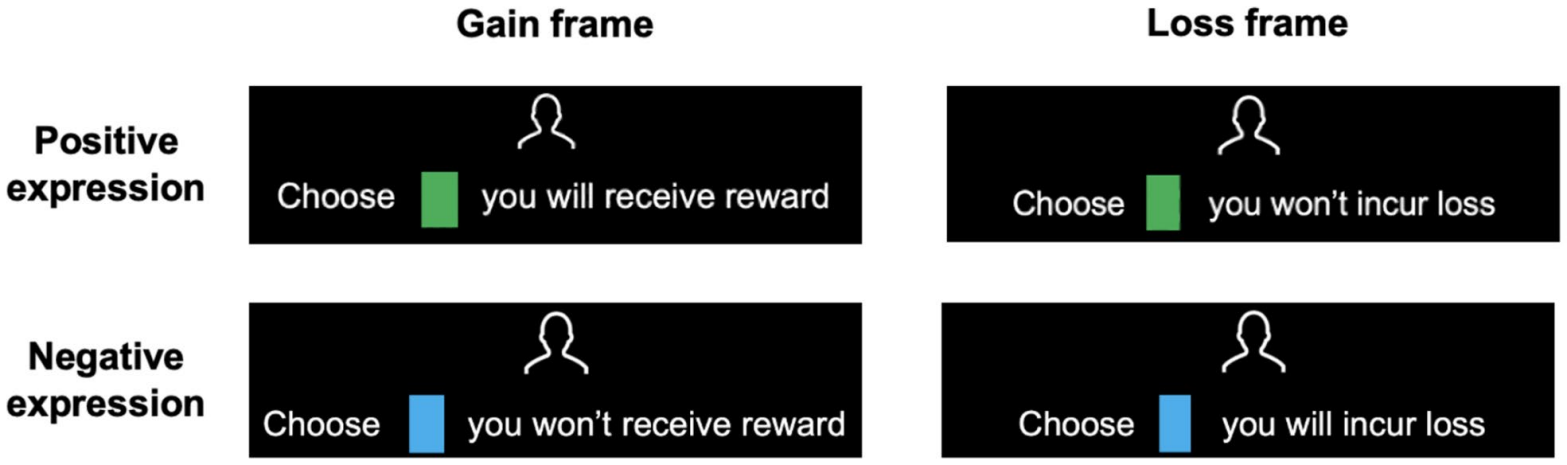
Advice examples in Exp 2. Participants were assigned to either a gain frame or a loss frame. In the gain frame, advice focuses on whether the participant will receive a reward, while in the loss frame, advice focuses on whether the participant will incur a loss.

The procedure for Experiment 2 was essentially the same as experiment 1. It is important to note that before the experiment, participants were randomly assigned to one of two task frames. In the gain frame, participants received a reward for making the correct choice and no reward for making the wrong choice in each trial. In the loss frame, each trial, participants incurred a loss for making the wrong choice and no loss for making the correct choice. In the gain frame, the positive expression of the advice was “Choose a card; you will receive a reward,” while the negative expression was “Choose a card; you will not receive a reward.” In the loss frame, the positive expression was “Choose a card; you will not incur loss,” while the negative expression was “Choose a card; you will incur a loss” (see Figure 6).

In the gain frame, the initial participant’s bonus payment was 10 yuan. When the red bar extends beyond the silver marker on the right, the payment increases by 3 yuan, and when it reaches the far right, the payment increases by 5 yuan. In the loss frame, the initial participant’s bonus payment was 15 yuan. When the red bar shortens to the silver marker on the left, the payment decreases by 3 yuan, and if the bar disappears, the payment decreases by 5 yuan.

#### Computational modeling

The modeling methods in experiment 2 are the same as in experiment 1. Eight different models were used to fit the behavioral data, followed by model comparison to identify the winning model. After determining the winning model, parameter analysis was conducted using the posterior mean of its parameter distribution.

To capture framing-related differences in subjective evaluation, we implemented the following value codings for self-learning and advice-learning: For self-learning, in the gain frame, $R_{t}^{c}$ = 1 when the participant’s choice was correct, and $R_{t}^{c}$ = 0 when it was incorrect. The initial expected value was set to ( $V{\left(i\right)}_{1}^{c}$ ) = 0.5, reflecting a neutral-to-positive baseline. In the loss frame, $R_{t}^{c}$ = 0 when the participant’s choice was correct, and $R_{t}^{c}$ = − 1 when it was incorrect. The initial expected value was set to ( $V{\left(i\right)}_{1}^{c}$ ) = −0.5 to reflect a neutral-negative starting point. For advice-learning, the reward ( $R_{t}^{a}$ ) was coded as 1 when the advice was correct, and −1 when it was incorrect. The initial expected values for advice content ( $V{\left(i\right)}_{1}^{a}$ ) were set to 0 at the start of the task. These frame-specific codings enabled the model to capture differential subjective interpretations of feedback in gain and loss contexts.

#### Statistical analysis

For behavioral data, a two-factor repeated-measures ANOVA was conducted to investigate whether expression and task frame had a significant impact on participants’ task performance and advice-choice consistency rate. For the model-based data, a two-factor repeated-measures ANOVA was conducted to investigate whether the advice reference weight *ω* and the advice learning rate (α) differed across expressions and task frames.

### Results

#### Behavioral results

*Accuracy*: The results revealed no significant main effect of advice phrasing on participants’ decision accuracy (*F*(1, 37) = 0.37, *p* = 0.546, η^2^ = 0.010, 95% CI [0.00, 0.13]). Similarly, there was no significant main effect of framing (F(1, 37) = 0.00, *p* = 0.982, η^2^ = 0.000, 95% CI [0.00, 0.13]). This indicates that neither the expression nor the task frame had an impact on the participants’ task performance.

**Figure 7:**
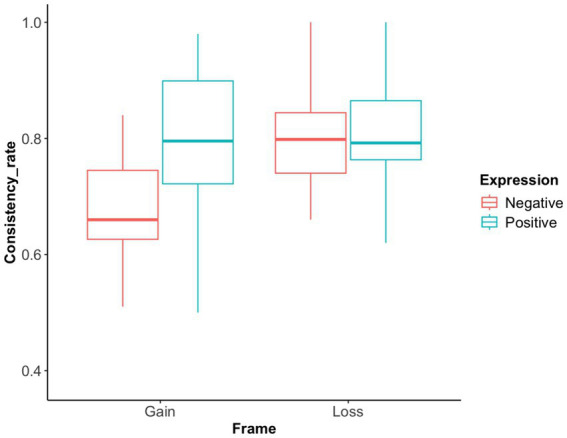
Advice-choice consistency rate in Exp 2. The advice-choice consistency rates under different frames and expressions were depicted. Generally, the consistency rate in the loss frame is significantly higher than in the gain frame. Positively expressed advice also shows a significantly higher consistency rate than negatively expressed advice. Furthermore, in the gain frame, the consistency rate for positive expressions is significantly higher than for negative expressions. However, in the loss frame, the consistency rate for negative expressions is higher than for positive expressions, though not significantly so.

*Advice-choice consistency rate*: The results revealed significant main effects for both the expression and task frame, as well as a significant interaction between the two factors (F(1, 37) = 7.47, *p* = 0.010, η^2^ = 0.168, 95% CI [0.03, 0.36]), as shown in Figure 7. The consistency rate for positively expressed advice was significantly higher than that for negatively expressed advice (F(1, 37) = 6.26, *p* = 0.017, η^2^ = 0.145, 95% CI [0.02, 0.33]). The consistency rate in the gain frame was significantly lower than that in the loss frame (F(1, 37) = 7.17, *p* = 0.011, η^2^ = 0.162, 95% CI [0.03, 0.35]). Further analysis showed that, in the gain frame, the consistency rate for positive expression was significantly higher than that for negative expression (t(18) = 3.66, *p* = 0.001, mean difference = 0.136, 95% CI [0.06, 0.21]). In the loss frame, the consistency rate was not significantly different between the two phrasing conditions (t(19) = 0.17, *p* = 0.868, mean difference = 0.006, 95% CI [−0.07, 0.08]). A Bayes factor (BF₁₀ = 0.24) further suggested limited evidence for a true effect.

#### Modeling results

Model comparison: The model comparison method used in this experiment is the same as in experiment 1. Results (see Table 2) showed that the winning model is still M4.

**Table 2:** Model comparison.

Categories	Model	Name	LOOIC
Basic RL	M1	ChoiceRL_AdviceRL	5316.0
Fict+RL	M2a	ChoiceFict_AdviceRL	5150.4
	M2b	ChoiceRL_AdviceFict	5107.4
	M2c	ChoiceFict_AdviceFict	5020.9
RL±	M3a	ChoiceRL±_AdviceRL	5038.4
	M3b	ChoiceRL_AdviceRL±	5315.0
	M3c	ChoiceRL±_AdviceRL±	5290.6
Full	M4	ChoiceFictRL±_AdviceFictRL	4862.4

**Figure 8:**
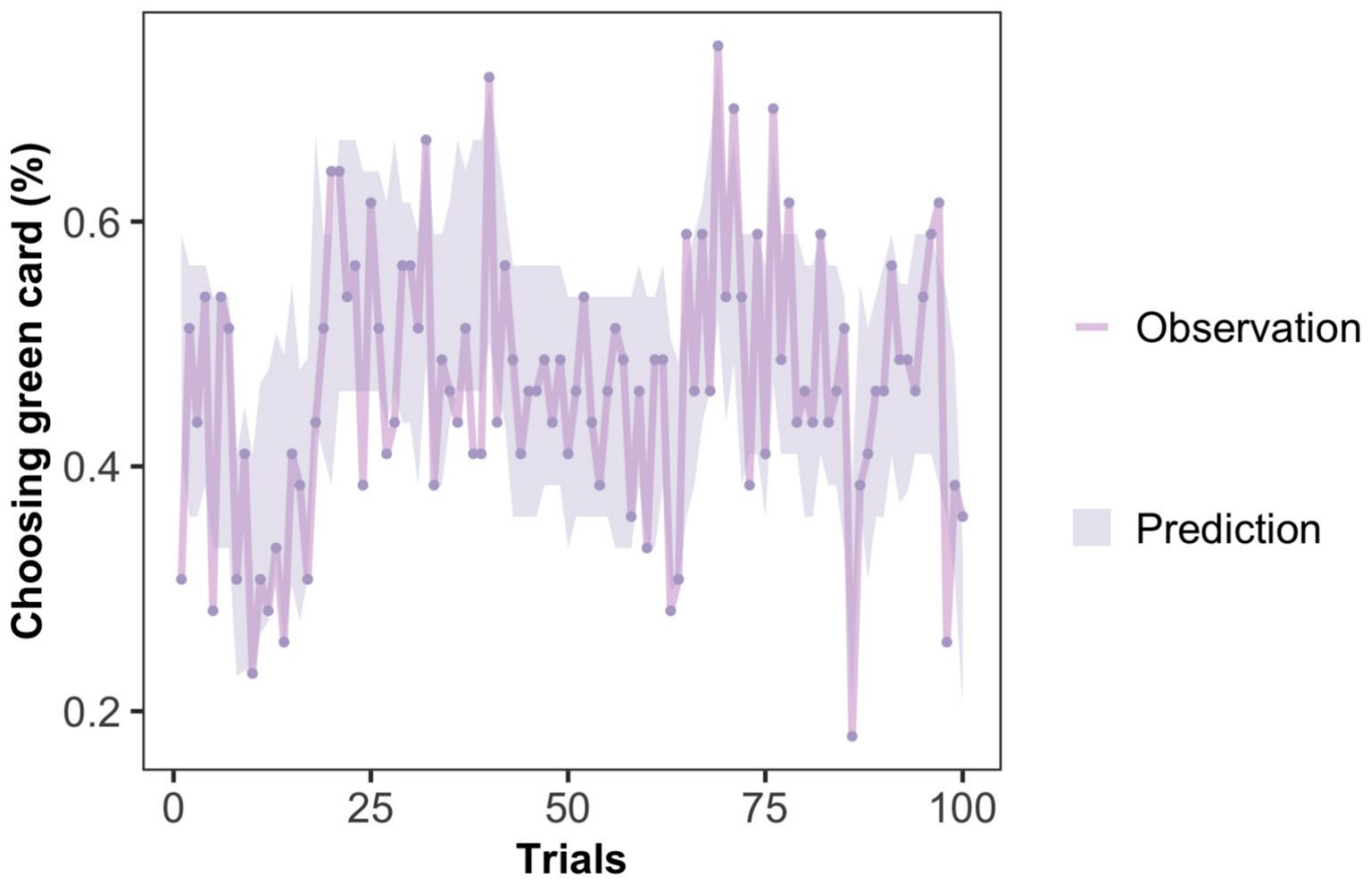
Posterior predictive check of winning model in Exp 2. The high overlap between the real and predicted data indicates that M4 captures the behavioral characteristics of the subjects in Exp 2.

*Model validation*: The same validation method was used, and the results are shown in Figure 8. There is a high degree of overlap between the predicted and observed data, indicating that M4 accurately captures the participants’ behavioral characteristics in experiment 2.

**Figure 9:**
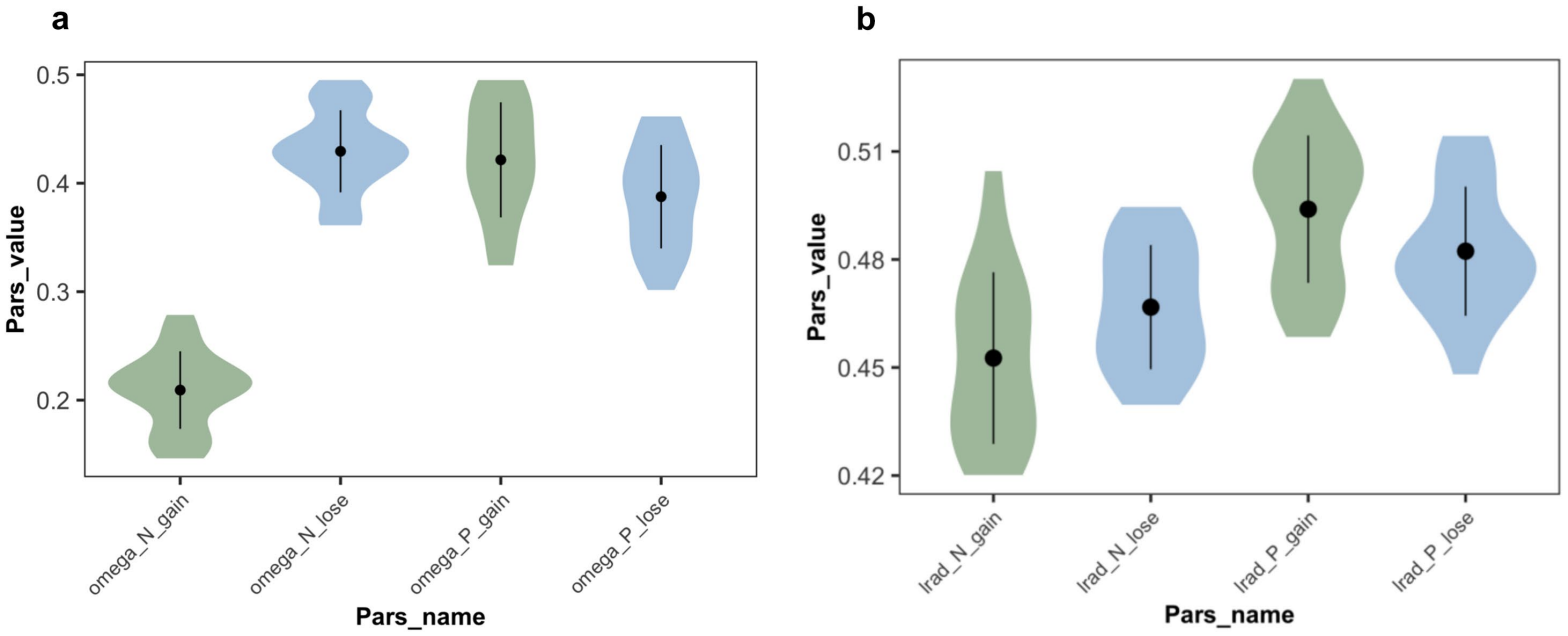
Estimated parameter values in Exp 2. **(a)** Weight parameters from the winning model, in which omega_P_gain and omega_N_gain represent the weight ω that subjects employed advice on trials associated with positive and negative expression in the gain frame. omega_P_lose and omega_N_lose represent the weight ω in the loss frame. Participants relied more on positively expressed advice than on negatively expressed advice when in a gain frame. However, in the loss frame, negatively expressed advice is more frequently used, though not significantly. **(b)** Learning rate parameters from the winning model, in which lrad_P_gain and lrad_N_gain represent the learning rate $\;\alpha^{a}$ of advice being correct associated with positive and negative expressions in the gain frame. lrad_P_lose and lrad_N_lose represent the learning rate in the loss frame. Same as Exp 1, the learning rate for positive expression was significantly higher than for negative expression. But there is no significant difference under task frames.

*Parameter analysis*: The statistical analysis results (see Figure 9a) showed significant main effects for both the expression and task frames, as well as a significant interaction between the two factors (*F*(1,37) = 148.61, *p* < 0.001, η^2^ = 0.686, 95% CI [0.56, 0.78]). The reference weight for positive expression was significantly higher than for negative expression (F(1,37) = 66.85, p < 0.001, η^2^ = 0.496, 95% CI [0.35, 0.62]). The reference weight in the gain frame was significantly lower than in the loss frame (F(1,37) = 84.58, *p* < 0.001, η^2^ = 0.540, 95% CI [0.40, 0.65]). Further analysis showed that, in the gain frame, the reference weight for positive expression was significantly higher than for negative expression (t(18) = 14.60, *p* < 0.001, mean difference = 0.212, 95% CI [0.18, 0.24]). In the loss frame, the reference weight for negative expression was significantly higher than for positive expression (t = 2.80, *p* = 0.008, mean difference = 0.042, 95% CI [0.01, 0.07]).

The statistical analysis results for the advice-taken learning rate $\alpha^{a}$ (see Figure 9b) showed a significant main effect of advice phrasing (F(1,37) = 38.44, *p* < 0.001, η^2^ = 0.347, 95% CI [0.22, 0.48]), no significant main effect of task frame (F(1,37) = 0.07, *p* = 0.791, η^2^ = 0.001, 95% CI [0.00, 0.027]), and a significant interaction between the two factors (F(1,37) = 7.91, *p* = 0.008, η^2^ = 0.099, 95% CI [0.02, 0.22]). Specifically, the learning rate for positive expression was significantly higher than for negative expression. This indicates that the phrase has a significant impact on the learning rate when advice is taken, whereas the task frame does not.

## Discussion

This study used two experiments to investigate the impact of different advice phrasing on individuals’ likelihood of taking advice and how this effect varies when the decision task is framed as a gain or a loss. Behavioral measures of advice-taking, such as the advice-choice consistency rate, have limitations, as selecting the same option as the advice does not necessarily indicate true adoption. To indirectly address this issue, we employed computational modeling to quantify the degree to which individuals reference advice (parameter ω ). By combining behavioral measures with computational modeling, this study provides more robust evidence supporting its conclusions.

### The impact of advice phrasing and decision frames on advice-taking

Experiment 1 examined the impact of two different types of advice phrasing on advice-taking when both gains and losses were possible. Both behavioral and modeling results indicated that individuals were more likely to take advice phrased positively than negatively, consistent with previous findings (Jang and Feng, 2018; Piray et al., 2021), thus supporting Hypothesis 1. Several studies in psychology, neuroscience, and behavioral economics have shown that human decision-making is often influenced by how a decision context is framed (Kahneman and Tversky, 1979; Kahneman et al., 1982; De Martino et al., 2006; Rangel et al., 2008). In the context of advice-taking, this influence manifests as a preference for positively framed advice. But how can this effect be explained? Tversky and Kahneman (1991) argued that when outcomes are presented positively, individuals tend to avoid potential risks, whereas when outcomes are presented negatively, individuals tend to seek risk. When presented with positively phrased advice (focusing on potential gains), participants, in an effort to avoid the risk of losing money, were more inclined to follow the advice (note that participants were informed prior to the experiment that the advisor’s accuracy was generally greater than 50%, making it less risky to follow the advice in the absence of a clear card pattern). Conversely, when presented with negatively phrased advice (focusing on potential losses), participants were more likely to take risks and choose the option opposite to the advice. Another possible explanation is that although both types of advice convey the same information, they require different levels of cognitive resources to process. Positive phrases are more straightforward, directing participants toward the card that the advice suggests they should choose, while the negative phrase requires participants to first “strip away” the negation before inferring the suggested option. This additional cognitive demand violates the cooperative principle in human communication (Grice, 1975), leading participants to be less inclined to adopt the advice.

Additionally, Study 1 found that advice phrasing influenced the learning rate for advice adherence, with individuals being more sensitive to changes in the accuracy of positively phrased advice. This finding may indirectly support the second explanation, suggesting that a positive phrase is processed more easily because of its lower cognitive demands, thereby making participants more responsive to changes in its accuracy. However, the first explanation cannot be entirely ruled out.

Experiment 2 built on experiment 1 by exploring the combined effects of advice phrasing and decision frames on advice-taking. The results showed that under a gain frame, individuals were more likely to follow positively phrased advice than negatively phrased advice. Under a loss frame, negatively phrased advice exerted a relatively stronger influence—most clearly reflected in the estimated advice weight (*ω*). However, this pattern was not seen at the behavioral level. This discrepancy may reflect the fact that the model-derived advice weight (ω) is potentially more sensitive to underlying decision processes than surface-level behavioral consistency. While advice-choice consistency reflects whether choices align with advice, ω captures the extent to which advice was actually weighted during the decision process—regardless of whether the final choice matched the advice.

Previous studies have yielded similar findings. For instance, Haddad and Delhomme (2006) found that under high-threat conditions, negatively phrased advice is more persuasive, whereas under low-risk conditions, positively phrased advice is more effective. Rothman et al. (2006) found that when preventing disease onset, advice emphasizing potential gains is more persuasive in promoting healthy behaviors, while after detecting a disease, advice emphasizing potential losses is more persuasive. The regulatory fit theory (Higgins, 2000) might explain this phenomenon. According to Higgins, individuals exhibit specific tendencies—promotion orientation (seeking opportunities) or prevention orientation (avoiding risks)—when pursuing goals. When an individual’s regulatory orientation matches the way information is framed, it influences their decision-making behavior (Avnet and Higgins, 2006). In the context of advice-taking, a substantial body of research suggests that such a regulatory fit promotes advice-taking (Bosone et al., 2015; Van Kleef et al., 2015; Du et al., 2022). Regulatory orientation can be triggered by specific task contexts, with a promotion orientation being activated in gain contexts and a prevention orientation in loss contexts (Motyka et al., 2014). According to this theory, in the current study, participants with a promotion orientation activated under a gain frame achieved regulatory fit when they received positively phrased advice (emphasizing opportunity-seeking), which enhanced their tendency to follow the advice. This matching effect resembles egocentric bias, as individuals are more inclined to trust advice that aligns with their activated regulatory orientation. Moreover, some researchers suggest that when an individual’s goal-pursuit strategy aligns with the information frame, the processing of that information becomes more fluent, thereby enhancing its evaluation (Lee and Aaker, 2004). Other scholars have focused on the perceived value of the information, finding that when such a match occurs, participants assign higher value to the information, which in turn strengthens their tendency to adopt the advice (Wang et al., 2011). In other words, when the decision frame matches the advice phrasing, participants may attribute greater value to the advice, making them more likely to adopt it. In this study, the advice reference weight ω can also be interpreted as a measure of trust in the advisor, suggesting that regulatory fit may enhance participants’ trust in the advisor, thereby increasing their likelihood of following the advice.

Additionally, the optimal model in this study assumed that participants employed a fictitious reinforcement learning strategy in both learning the card accuracy and learning the advice accuracy, meaning they updated the value of both chosen and unchosen options (followed and unfollowed advice) simultaneously. The model also assumed that participants exhibited asymmetrical learning rates when learning the card accuracy, but not when learning the advice accuracy. Lefebvre et al. (2017) suggested that asymmetrical learning rates reflect individuals’ positive biases (i.e., selectively processing information that supports their beliefs) and that these biases exist in “lower-level” reinforcement learning processes. This might suggest that, compared to processing the advice, individuals use a simpler processing strategy when learning card accuracy—selectively processing information that aligns with their expectations.

### Biases in processing social information

Individuals exhibit biases when processing social information. Variations in advice-taking across different phrasings indicate that decision-making using social information is easily influenced by how the information is framed. This influence is also evident in everyday consumer behavior. For example, Tiffany et al. (2020) investigated how to effectively reduce shopping cart abandonment rates and found that for functional products, messages emphasizing benefits were more effective than those emphasizing losses. Such biases in processing social information manifest in various ways, including egocentric bias (i.e., the tendency to rely more on personal information) (Heyes, 2016; Samuel, 2023), confirmation bias (i.e., the tendency to undervalue social information that contradicts one’s initial judgment) (Kappes et al., 2020), and reciprocity bias (i.e., the weight we give to others’ advice is influenced by the weight they give to our advice) (Mahmoodi et al., 2018; Zonca et al., 2021). In the context of advice-taking explored in this study, participants need to overcome egocentric bias by fairly considering both self-generated and external information. Additionally, they must address confirmation bias by evaluating advice equally, even when it contradicts their expectations.

### Contributions, limitations, and future directions

Previous research on factors influencing advice taking often focuses on a single aspect. However, in real interpersonal communication, advice-taking is simultaneously influenced by the decision-maker, the advisor, the advice characteristics, and the decision task. This study simultaneously considers the effects of advice characteristics, decision tasks, and their interaction on advice taking, thereby enriching the existing research on factors influencing advice adoption. Additionally, the experimental paradigm used in this study ensures that the advice information conveyed by the two phrasing types is equivalent (e.g., “Choose the green card, you will receive a reward” versus “Choose the blue card, you will incur a loss”), so that differences in advice taking can be attributed solely to the phrasing. This study also employs cognitive modeling to quantify advice-taking behavior as advice reference weight, providing evidence from a cognitive perspective on how decision frames and advice phrasing affect advice-taking. Moreover, the study has practical implications, offering insights into how advice phrasing affects advice adoption in various task contexts. For example, in financial consulting before an investment decision, advisors could emphasize gains rather than potential losses to influence decision-makers, while decision-makers should avoid cognitive biases, such as egocentric bias, to make more rational decisions.

Despite its contributions, this study has several limitations. First, real-life decisions often involve multiple options, where the informational value of positive versus negative advice may differ. For instance, highlighting a product’s strengths may directly suggest a choice, while pointing out weaknesses may only help eliminate options. Our binary-choice design did not capture this complexity, and future work could extend the paradigm to multi-option scenarios to improve ecological validity. Second, the adviser was simulated as a computer with stable, helpful intentions and fixed accuracy. In reality, advisers may hold varying or even malicious intentions, which can undermine trust and affect advice-taking. Future research could explore how such intentional volatility shapes learning and decision-making. Third, we modeled the advice weight (*ω*) as a fixed trait-level parameter. More dynamic models—such as those incorporating state-based advice evaluation or Bayesian updates—may better capture individual learning trajectories and should be tested in future work. Finally, two of the four advice conditions used grammatical negation (e.g., “you will not incur loss,” “you will not receive reward”), potentially confounding motivational valence with syntactic form. While our expressions mirrored real-life phrasing, future studies could adopt syntactically balanced alternatives (e.g., “you will avoid loss,” “you will miss reward”) to isolate effects of framing from those of linguistic structure.

Furthermore, future studies could investigate the neural mechanisms underlying advice taking. For instance, the vmPFC has been shown to integrate value-based decision-making with reward and social information (Behrens et al., 2008), a function that appears similar to the advice reference weight in this study. Future research could explore whether activity in this brain region correlates with advice reference weight and how various factors might influence this brain activity, thereby affecting advice taking.

## Conclusion

Advice phrasing significantly affects advice referencing. When both gains and losses are possible, individuals are more likely to trust and take advice when it is presented positively.

Under a gain frame, individuals are more likely to reference positively phrased advice; under a loss frame, they are more likely to reference negatively phrased advice.

## Data Availability

The original contributions presented in the study are included in the article/Supplementary material; further inquiries can be directed to the corresponding author.
