# Supplementary material for: The impact of phrasing on advice-taking under gain and loss frames in a reinforcement learning paradigm
Source: Front Psychol. 2025 Dec 17;16:1693546. doi: 10.3389/fpsyg.2025.1693546 (PMC12753883; doi:10.3389/fpsyg.2025.1693546)
Supplement: Supplementary file 1 [file Table_1.DOCX]

**Supplementary**

**Participant Instructions**

**Experiment 1:**

Welcome, and thank you for participating in our experiment.

You will be taking part in a card-based task. On each trial, a green card and a blue card will appear on the screen. Your goal is to guess which card hides a reward by pressing the “F” or “J” key to make your selection. **If you choose the correct card, you will receive a reward; if you choose incorrectly, you will incur a loss**.

During the task, you will receive advice from a computer-generated advisor who has access to more information than you do. Their advice is correct more than 50% of the time. However, please note that the suggestions may not always be accurate. You can decide whether or not to follow the advice on each trial.

At the bottom of the screen, you will see a progress bar that shows your accumulated earnings. **If the red bar extends beyond the silver marker on the right, your participation bonus will increase by 3 yuan. Conversely, if the red bar shrinks below the silver marker on the left, your bonus will decrease by 2 yuan**. Your base participation payment is 12 yuan.

If you understand the task instructions, please press the “Q” key to begin the practice trials.

**Experiment 2:**

**Gain frame:**

Welcome, and thank you for participating in our experiment.

You will be taking part in a card-based task. On each trial, a green card and a blue card will appear on the screen. Your goal is to guess **which card hides a reward** by pressing the “F” or “J” key to make your selection. **If you choose the correct card, you will receive a reward; if you choose incorrectly, you will not receive a reward**.

During the task, you will receive advice from a computer-generated advisor who has access to more information than you do. Their advice is correct more than 50% of the time. However, please note that the suggestions may not always be accurate. You can decide whether or not to follow the advice on each trial.

At the bottom of the screen, you will see a progress bar that shows your accumulated earnings. **If the red bar extends beyond the silver marker on the right, your participation bonus will increase by 3 yuan. If it reaches the red marker, your bonus will increase by 5 yuan.** Your base participation payment is 10 yuan.

If you understand the task instructions, please press the “Q” key to begin the practice trials.

**Loss frame:**

Welcome, and thank you for participating in our experiment.

You will be taking part in a card-based task. On each trial, a green card and a blue card will appear on the screen. Your goal is to guess which card does not contain a trap by pressing the “F” or “J” key to make your selection. **If you choose the incorrect card, you will incur a loss; if you choose correctly, you will not incur a loss**.

During the task, you will receive advice from a computer-generated advisor who has access to more information than you do. Their advice is correct more than 50% of the time. However, please note that the suggestions may not always be accurate. You can decide whether or not to follow the advice on each trial.

At the bottom of the screen, you will see a progress bar that shows your accumulated earnings. **If the red bar shrinks below the silver marker on the left, your participation bonus will decrease by 3 yuan. If it shrinks to the gold marker, your base payment will decrease by 5 yuan.** Your base participation payment is 15 yuan.

If you understand the task instructions, please press the “Q” key to begin the practice trials.

**Advice Phrasing Assignment**

In each trial, the content of the advice (i.e., which card to choose) was first determined according to a predefined probabilistic schedule, with the advisor’s overall accuracy fixed at 60%. Once the intended advice direction was determined (e.g., “choose green”), the phrasing of the advice—positive or negative—was randomly assigned.

Specifically, the 100 experimental trials were divided into 10 blocks of 10 trials. Within each block, 5 trials were randomly assigned to the positive phrasing condition (e.g., “Choose the green card, you will receive reward”) and 5 to the negative phrasing condition (e.g., “Choose the blue card, you will incur loss”). This ensured a balanced number of positively and negatively phrased advice trials across the experiment (50/50 ratio). The phrasing assignment was orthogonal to advice correctness and to the spatial position of the cards.

**Construction of Models**

**Fict+RL Models**

These models assume that participants' learning of card accuracy (advice accuracy) follows the principles of Fictitious Reinforcement Learning, where value updates for both chosen card (following advice) and unchosen card (not following advice). For example, the ChoiceFict_AdviceFict model, which assumes that participants' learning of both card accuracy and advice accuracy follows Fictitious Reinforcement Learning. Its cognitive model is as follows:

Self-learning:

Chosen card: ${V(i)}_{t+1}^{c}$=${V(i)}_{t}^{c}+\alpha^{c}$ · $(R_{t}^{c}-{V(i)}_{t}^{c})$

Unchosen card：${V(i)}_{t+1}^{cn}$=${V(i)}_{t}^{cn}+\alpha^{cn}$ · $({-R}_{t}^{c}-{V(i)}_{t}^{cn})$

Advice-learning：

Following advice： ${V(i)}_{t+1}^{a}$=${V(i)}_{t}^{a}+\alpha^{a}$ · $(R_{t}^{a}-{V(i)}_{t}^{a})$

Not following advice：${V(i)}_{t+1}^{an}$=${V(i)}_{t}^{an}+\alpha^{an}$ · $({-R}_{t}^{a}-{V(i)}_{t}^{an})$

Here, *c* and *cn* represent learning from chosen and unchosen cards, while *a* and *an* represent learning from following and not following advice.

The modeling for the ChoiceFict_AdviceRL and ChoiceRL_AdviceFict models is similar to the above. The former updates values for chosen and unchosen options in card learning, while the latter updates values for following and not following advice in advice learning.

**RL±Models**

These models assume that participants exhibit asymmetric learning rates when learning card accuracy (advice accuracy), meaning that the learning rates differ depending on whether the prediction error is positive or negative. For the ChoiceRL±_AdviceRL± model, which assumes asymmetric learning rates in both self-learning and advice-learning. Its cognitive model is as follows:

Self-learning:

${V(i)}_{t+1}^{c}$=${V(i)}_{t}^{c}+a^{c+}$ · ${PE}_{t}^{c}$ (${PE}_{t}^{c}$ ≧ 0)

${V(i)}_{t+1}^{c}$=${V(i)}_{t}^{c}+a^{c-}$ · ${PE}_{t}^{c}$ (${PE}_{t}^{c}$ < 0)

Advice-learning:

${V(i)}_{t+1}^{a}$=${V(i)}_{t}^{a}+a^{a+}$ · ${PE}_{t}^{a}$ (${PE}_{t}^{a}$ ≧ 0)

${V(i)}_{t+1}^{a}$=${V(i)}_{t}^{a}+a^{a-}$ · ${PE}_{t}^{a}$ (${PE}_{t}^{a}$ < 0)

Here, + and – differentiate between learning rates when the prediction error is positive and negative. The modeling process for the ChoiceRL±_AdviceRL and ChoiceRL_AdviceRL± models is similar to the above.

**M4 Model**

The ChoiceFictRL±_AdviceFictRL model combines the winning models from the previous two types: ChoiceFict_AdviceFict and ChoiceRL±_AdviceRL. It assumes that participants' self-learning follows Fictitious Learning and distinguishes between two learning rates, while their advice-learning also follows Fictitious Learning without distinguishing between the two learning rates. The modeling process is not elaborated here.
